# Role of the linker region in the expression of *Rhizopus oryzae *glucoamylase

**DOI:** 10.1186/1471-2091-8-9

**Published:** 2007-06-25

**Authors:** Shu-Chuan Lin, Wei-Ting Liu, Shi-Hwei Liu, Wei-I Chou, Bor-Kai Hsiung, I-Ping Lin, Chia-Chin Sheu, Margaret Dah-Tsyr Chang

**Affiliations:** 1 Institute of Molecular and Cellular Biology & Department of Life Science, National Tsing Hua University, Hsinchu, Taiwan 300, Republic of China; 2 Simpson Biotech Co., Ltd, Taoyuan Country, Taiwan 333, Republic of China

## Abstract

**Background:**

*Rhizopus oryzae *glucoamylase (*Ro*GA) consists of three domains: an amino (N)-terminal raw starch-binding domain (SBD), a glycosylated linker domain, and a carboxy (C)-terminal catalytic domain. The 36-amino-acid linker region (residues 132–167) connects the two functional domains, but its structural and functional roles are unclear.

**Results:**

To characterize the linker sequences of *Ro*GA and its involvement in protein expression, a number of *Ro*GA variants containing deletions and mutations were constructed and expressed in *Saccharomyces cerevisiae*. Deletion analyses demonstrate that the linker region, especially within residues 161 to 167, is required for protein expression. In addition, site-directed mutagenesis and deglycosylation studies reveal that the linker region of *Ro*GA contains both N- and O-linked carbohydrate moieties, and the N-linked oligosaccharides play a major role in the formation of active enzyme. Although the linker segment itself appears to have no ordered secondary structural conformation, the flexible region indeed contributes to the stabilization of functional N- and C-terminal domains.

**Conclusion:**

Our data provide direct evidence that the length, composition, and glycosylation of the interdomain linker play a central role in the structure and function of *Ro*GA.

## Background

Glucoamylase (1,4-α-D-glucan glucohydrolase, EC 3.2.1.3; GA) is an exohydrolase that catalyzes the release of β-D-glucose by hydrolyzing α-1,4- and α-1,6-glucosidic linkages at the non-reducing ends of raw or soluble starches and related oligosaccharides [1-3]. GA has been used in industrial processes such as the production of glucose syrup and other food-processing applications [2,4]. Although many fungal species are capable of producing GA under different growth conditions [5], the industrial development of GA has focused only on GA from *Aspergillus niger *(*An*GA; identical to *Aspergillus awamori *GA) and *Rhizopus oryzae *(*Ro*GA) because of their stability and high activity [3,6,7]. The overall domain structure of *An*GA consists of an N-terminal catalytic region and a C-terminal starch-binding domain (SBD). In contrast, the organization of that of the *Ro*GA consists of an N-terminal SBD and a C-terminal catalytic region. The biochemical properties of *An*GA have been well characterized [2,8-16], whereas less is known about *Ro*GA [17,18]. This work focuses on functional analysis of *Ro*GA.

**Figure 1:**
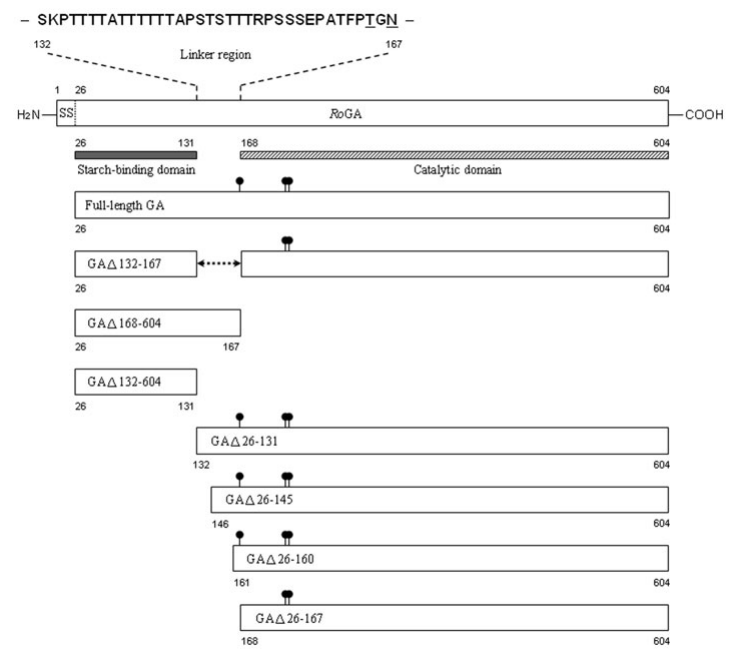
**Schematic representation of the wild-type *Ro*GA and various mutant of *Ro*GA**. Full-length *Ro*GA and its deletion mutants were cloned into the yeast expression plasmid pS1. Each construct was designed to have a natural signal sequence (SS) for secretion. The starch-binding and catalytic domains and the sequence of the linker region (amino acids 132–167) of *Ro*GA are indicated. The residues in the linker which were subjected to mutagenesis are underlined. Closed circles indicate the potential N-linked glycosylation sites.

*Ro*GA is synthesized as a precursor containing a typical hydrophobic secretory signal sequence of 25 amino acids. The mature form of *Ro*GA is a single-chain protein composed of three domains: an SBD (residues 26–131), a Thr/Ser-rich linker region (residues 132–167), and a catalytic domain (residues 168–604) [19]. The schematic representation of *Ro*GA is shown in Figure 1. TheN-terminal SBD belongs to the carbohydrate-binding module (CBM) family 21 and shows a relatively low level of similarity to SBDs in other starch-degrading enzymes [20-23]. The C-terminal catalytic domain of *Ro*GA plays an active role in hydrolyzing starch and has a high degree of sequence similarity to those of other fungal GAs [6]. The linker region between these two functional domains is rich in hydroxyl-amino acid residues, but information about its function is quite limited [16,24,25]. In the CAZy classification based on amino acid sequences of the catalytic domain, GAs are classified into glycoside hydrolase (GH) family 15 [26], and on two recently published articles [27,28], the N-terminally positioned SBD (CBM21) and the C-terminally positioned SBD (CBM20) are classified to be grouped into a common CBM clan. However, the sequences of the linker regions are highly variable. Comparison of the primary structures of different fungal GAs reveals that the linker sequences vary greatly in length and composition (Table 1).

In some fungal hydrolases, the substrate-binding and catalytic domains are separated by a linker segment rich in proline and hydroxy amino acid residues, some of which have been shown to be involved in various functions including enzymatic activity, stability, protein secretion, and ligand binding [29,30]. In *An*GA, the heavily O-glycosylated linker domain is essential for secretion and is responsible for enzyme stability as well as activity toward raw starch [11,16,24]. Aside from the knowledge that the linker region in *Ro*GA acts as an interdomain spacer, very little is known about this specific sequence stretch. The linker region of *Ro*GA contains high percentages of Thr (44%) and Ser (16%) residues. Along with numerous putative O-glycosylation sites, the *Ro*GA linker also contains one potential N-glycosylation site adjacent to the catalytic domain (Asn^167^-Ser-Thr, Figure 1).

**Table 1:** Amino acid sequences of the linker region from fungal GAs. Potential glycosylation sites in the linker sequences were predicted by the NetNGlyc and NetOGlyc programs. The predicted N-glycosylation sites are underlined and the putative O-glycosylation sites are labeled in bold.

GA*	**Position (aa)**	**Linker sequence**
*Ro*GA	132–167	SKP**TTTT**A**TTTTTT**AP**STSTTT**RP**SSS**EPA**T**FP**T**GN
*An*GA	494–531	A**T**GG**TTTT**A**T**P**T**G**S**G**S**V**TSTS**K**TT**A**T**A**S**K**TSTSTSSTS**
*Hr*GA	500–504	NVTSS
*Hg*GA	490–508	**S**KQ**T**PNP**S**AAP**S**P**S**PYP**T**A
*Mc*GA	129–177	SV**TTTTTT**AP**TTTTS**GG**SSTTT**GG**STTT**A**TS**VP**T**GVPSGFP**T**GNS**T**ISS
*Nc*GA	494–519	A**T**A**T**A**TS**FPANL**T**PA**STT**V**T**PP**T**QTG

*GenBank or GenPept accession numbers are as follows: *Ro*, *Rhizopus oryzae *(from Simpson Biotech); *An*, *Aspergillus niger *(AAP04499); *Hr*, *Hormoconis resinae *(Q03045); *Hg*, *Humicola grisea *(AAA33386); *Mc*, *Mucor circinelloides *(AAN85206); and *Nc*, *Neurospora crassa *(P14804).

Here the baker's yeast, *Saccharomyces cerevisiae*, was used as a host system to study the structure and function of *Ro*GA, with special attention to the interdomain linker region. The effects of the linker segment with specific length, composition, and glycosylation on the properties of the protein expression, ligand or substrate binding, enzyme activity and stability have been thoroughly investigated.

## Results and Discussion

### Expression of the full-length and deletion mutant GAs in S. cerevisiae

**Figure 2:**
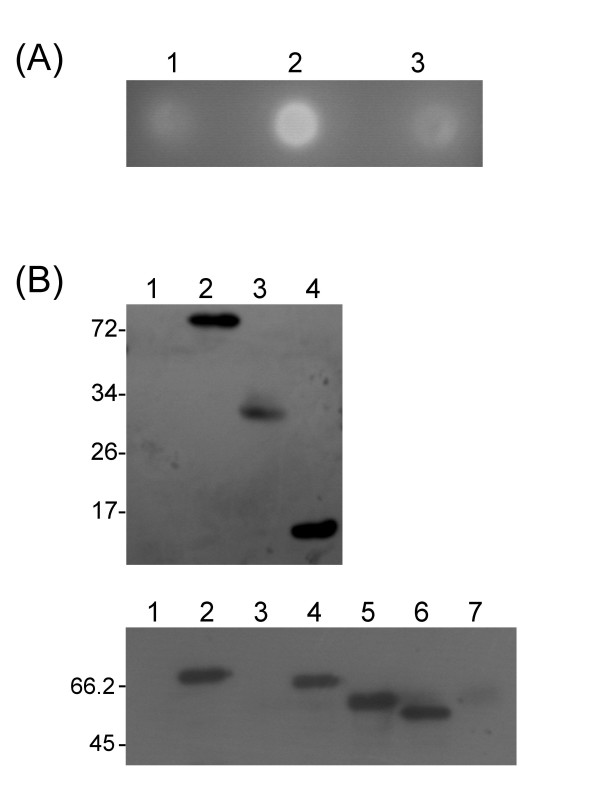
**Expression of recombinant *Ro*GAs in Saccharomyces**. (A) Starch plate assay for GA expression in 10 μL (*A*_600 _= 2) *S. cerevisiae *cells carrying pS1 (lane 1), pS1/full-length GA (lane 2) and pS1/GAΔ132–167 (lane 3) plasmids growth on a SD plate containing 0.5% soluble starch at 30°C and stained with 0.01% iodine solution. (B) Yeast strain was transformed with expression plasmids containing the full-length or mutant GA insert. After incubation at 30°C for 3 days, the supernatants were collected and concentrated. Culture supernatant (10 μL) from each clone was examined by Western blotting using anti-GA antibody. Upper panel, lane 1, vector only; lane 2, full-length GA; lane 3, GAΔ168–604 and lane 4, GAΔ132–604; lower panel, lane 1, vector only; lane 2, full-length GA; lane 3, GAΔ132–167; lane 4, GAΔ26–131; lane 5, GAΔ26–145; lane 6, GAΔ26–160 and lane 7, GAΔ26–167.

To investigate the function of the linker region of *Ro*GA, several constructs containing full-length, truncated or partially deleted GA fragments were generated (Figure 1). All constructs used in this study contained a leader signal sequence of the N-terminal 25 residues of *Ro*GA. When the full-length GA was expressed in *S. cerevisiae*, secreted enzyme activity was detected in the culture medium following incubation at 30°C (Figure 2A, lane 2 and 2B, lower panel, lane 2). In contrast, removal of the interdomain 36-amino acid linker of *Ro*GA (GAΔ132–167) led to generation of a mutant with no detectable secreted GA as determined by plate assay or Western blot analysis (Figure 2A, lane 3 and 2B, lower panel, lane 3), suggesting that the linker played an important role in the function of *Ro*GA. To further examine whether the linker region was essential for the formation of active starch-binding or catalytic domains, four deletion mutants devoid of either the substrate binding or catalytic domain in the presence or absence of the linker region were engineered to generate plasmids pS1-GAΔ168–604 encoding the SBD with the C-terminal linker region; pS1-GAΔ132–604 encoding the SBD alone; pS1-GAΔ26–131 encoding the linker region preceding the catalytic domain; and pS1-GAΔ26–167 encoding the catalytic domain only. It was found that two SBD-containing clones, one, GAΔ168–604, containing the linker sequence and the other, GAΔ132–604, without the linker sequence were both successfully expressed and secreted (Figure 2B, upper panel, lanes 3 and 4). Since no effect was observed in the absence of the linker in both cases, the linker region appeared to be not essential for the formation and secretion of the SBD. As for the catalytic domain-containing variants, GAΔ26–131 was successfully expressed and secreted in yeast, but GAΔ26–167 lacking of the linker sequence was not expressed at all (Figure 2B, lower panel, lanes 4 and 7), indicating that the linker region was specifically required for formation and secretion of the catalytic domain. To further define which region in the linker was crucial for its role in facilitating formation and secretion of the catalytic domain, several constructs encoding partial deletions of the linker region fused to the catalytic domain were subsequently expressed and characterized. Interestingly, both GAΔ26–145 and GAΔ26–160, unlike GAΔ26–167, were successfully expressed in secretory forms (Figure 2B, lower panel, lanes 5 and 6), indicating that the linker region from residues 161–167 was essential for *in vivo *folding and secretion of the active catalytic domain. It is of interest that a recently published article [7] on *Rhizopus oryzar *found a second GA gene, *amyB*, lacks the N-terminal SBD. The *amyB *gene contains the conserved residues in the catalytic domain important for starch hydrolysis, and the gene also contains a stretch of linker sequence (33 bp) preceding the catalytic domain. This finding also indicates that the small region of linker plays an important role in function of *Ro*GA.

### Characterization of recombinant GAs

**Figure 3:**
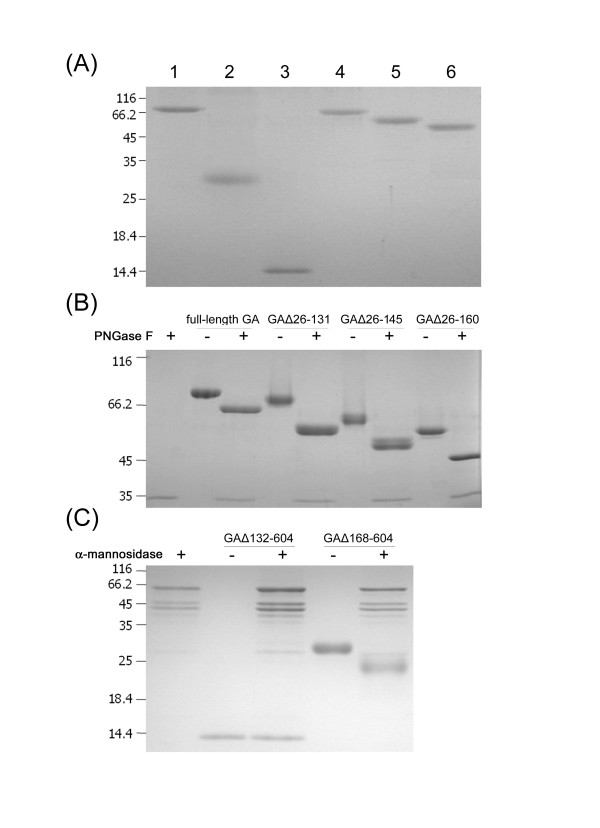
**Purification and deglycosylation of recombinant *Ro*GAs**. (A) Purified proteins (1 μg) obtained by cation-exchange chromatography were subjected to electrophoresis on a 15% SDS-PAGE and stained with Coomassie Brilliant Blue R-250. Lane 1, full-length GA; lane 2, GAΔ168–604; lane 3, GAΔ132–604; lane 4, GAΔ26–131; lane 5, GAΔ26–145 and lane 6, GAΔ26–160. (B) Protein samples were treated with PNGase F (250 U) and resolved by 10% SDS-PAGE. Symbols (+) and (-) indicate treatment with and without PNGase F, respectively. (C) Recombinant SBD was treated with Jack bean α-mannosidase and examined by 15% SDS-PAGE. Symbols (+) and (-) indicate treatment with and without Jack bean α-mannosidase, respectively.

In order to characterize *Ro*GA and to study the function of the linker region, the recombinant proteins including full-length GA, GAΔ168–604, GAΔ132–604, GAΔ26–131, GAΔ26–145, and GAΔ26–160 were individually expressed in the budding yeast *S. cerevisiae *and were grown in SD media at 30°C. Extracellular (secreted) proteins were concentrated from culture supernatants and purified by cation-exchange chromatography. As shown in Figure 3A, purified full-length GA, GAΔ168–604, GAΔ132–604, GAΔ26–131, GAΔ26–145, and GAΔ26–160 exhibited single protein bands with purity higher than 90%. SDS-PAGE and/or mass spectrometric determination revealed that the respective molecular mass of full-length GA, GAΔ168–604, GAΔ132–604, GAΔ26–131, GAΔ26–145, and GAΔ26–160 was approximately 78, 21, 12, 69, 60, and 56 kDa (Table 2). The differences between the calculated and observed molecular weights ranged from 3% to 28%, indicating that post-translational modifications occurred in some of the recombinant proteins, presumably due to differential degree of glycosylation. There are two main types of protein glycosylation: N-glycosylation and O-glycosylation. The former refers to the attachment of oligosaccharides to a protein through the amide of asparagine residues, whereas the latter involves attachment of sugars to the hydroxyamino acids serine and threonine *via *their hydroxyl groups [31]. Potential N-glycosylation sites in *Ro*GA analyzed using the on-line prediction server NetNGlyc version 1.0 [32] revealed that *Ro*GA contained three putative N-linked glycosylation consensus sites, one (Asn^167^-Ser-Thr) located in the linker region and two (Asn^230^-Thr-Thr and Asn^236^-Lys-Thr) in the catalytic domain (Figure 1). Although no specific consensus sequence for O-linked glycosylation has yet been reported [33,34], O-glycosylation may occur on any of the Ser or Thr residues within a short peptide region [35]. Interestingly, the program NetOglyc version 3.1 [36] predicted that potential O-glycosylation sites in *Ro*GA were clustered only in the linker region. Of the residues in the 36-amino-acid linker region, 61% were Ser and Thr, among which some might serve as potential sites for O-linked glycosylation. MALDI fingerprint mass spectrum from the tryptic digestion fragments of *Ro*GA (data not shown) also showed that no peptide fragment was mapped to the linker region, presumably due to the presence of heterogeneous glycosylation within this region.

**Table 2:** Comparison between calculated and experimental molecular weights of GAs. The molecular masses were calculated from the deduced amino acid compositions using the ExPASy Molecular Biology Server. The experimental molecular masses were obtained from mass spectrometry or estimated from SDS-PAGE.

**Protein**	**Calculated mass (kDa)**	**Apparent mass (kDa) (mass spectrometry/SDS-PAGE)**
full-length GA	62.4	ND*/78
T165A	62.4	ND*/78
N167D	62.3	ND*/76
GAΔ168–604	15.2	20.7/27
GAΔ132–604	11.7	12.0/14
GAΔ26–131	50.7	ND*/69
GAΔ26–145	49.4	59.9 to 61.1^†^/60
GAΔ26–160	47.9	55.5 to 56.6^‡^/55

*ND, not determined. ^†^GAΔ26–145 displayed multiple mass spectral peaks ranging from 59.9 to 61.1 kDa. ^‡^GAΔ26–160 displayed multiple mass spectral peaks ranging from 55.5 to 56.6 kDa.

To determine how much of the molecular weight discrepancy for each recombinant protein was derived from the addition of glycosyl groups, each protein was enzymatically deglycosylated by PNGase F or Jack bean α-mannosidase followed by SDS-PAGE analysis. PNGase F is an amidase that cleaves between the innermost GlcNAc and the asparagine residue of complex N-linked oligosaccharides from glycoproteins [37]. The fully glycosylated *Ro*GA possessed a molecular mass of 78 kDa. After removal of N-linked carbohydrates with PNGase F, its molecular mass was reduced to 65 kDa (Figure 3B), indicating that *Ro*GA produced by *S. cerevisiae *was highly glycosylated and the N-linked carbohydrates contributed approximately 13 kDa to the molecular weight. Enzymatic deglycosylation of asparagine-linked glycans in the catalytic domain variants GAΔ26–131, GAΔ26–145, and GAΔ26–160 also showed that these proteins were efficiently glycosylated to various degrees (Figure 3B).

The linker sequences code for a 36 amino acid extension at the C-terminal end of GAΔ168–604, which correspond to a molecular mass of approximately 3.5 kDa. The difference in molecular mass between GAΔ168–604 and GAΔ132–604 was 8.7 kDa, implying that the linker region contributed to the hyperglycosylation in GAΔ168–604. However, treatment of GAΔ132–604 and GAΔ168–604 with the N-glycan-specific PNGase F did not reduce its apparent molecular weight; hence there seemed to be little or no N-glycosylation on either protein (data not shown). At present, no enzyme comparable to PNGase F is available for removing intact O-linked sugars [38]. In general, O-glycans are short linear oligosaccharides consisting of one to five mannose residues in yeast [24,34,39]. Therefore, α-mannosidase, an exoglycosidase capable of removing terminal mannose, was used to treat the recombinant proteins. If O-linked carbohydrates were present, the overall mass of the protein would decrease after treatment with α-mannosidase. The SBD variant GAΔ132–604 did not respond to the treatment with α-mannosidase, such that no change was detected in the electrophoretic mobility (Figure 3C), suggesting that no major O-glycosylation occurred in this case. On the other hand, a clear shift in the mobility of GAΔ168–604 was observed after treatment with α-mannosidase, indicating that presence of the linker region led to a considerable degree of modification by high-mannose-type O-glycans (Figure 3C). Taken together, our data indicate that the linker region of *Ro*GA is modified by both N- and O-linked glycosylation, but the exact positions and functions are not clearly understood and require further investigation.

### Effects of mutations in the linker region of RoGA

**Figure 4:**
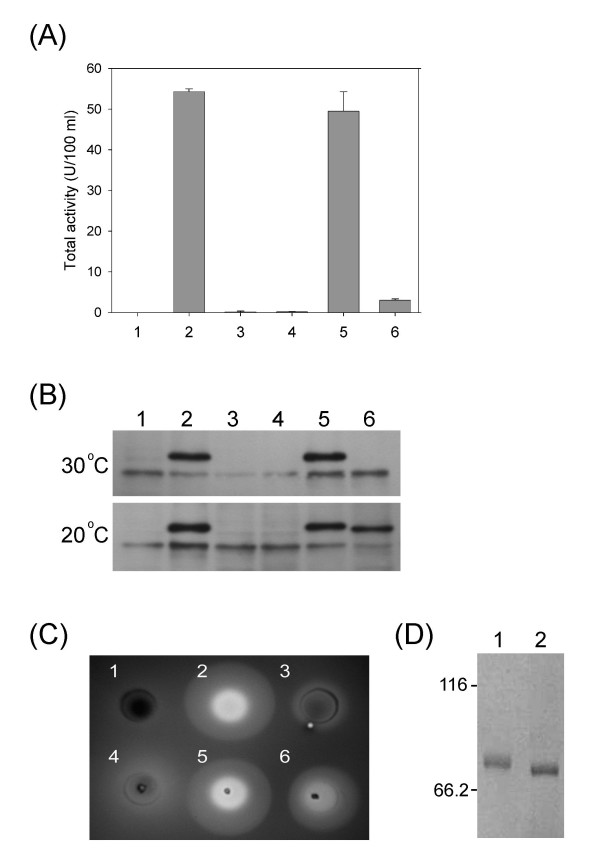
**Characterization of enzymatic function of recombinant *Ro*GAs**. (A) The extracellular activity of various recombinant GAs was tested. Lane 1, pS1 (empty vector); lane 2, full-length GA; lane 3, GAΔ26–167; lane 4, GAΔ132–167; lane 5, T165A and lane 6, N167D. (B) Western blot analysis of wild-type and mutant GAs grown at 30°C (upper panel) and 20°C (lower panel) for 3 days. Lane 1, pS1 (empty vector); lane 2, full-length GA; lane 3, GAΔ26–167; lane 4, GAΔ132–167; lane 5, T165A and lane 6, N167D. (C) Starch plate assays of mutant GAs. No. 1, *S. cerevisiae *MNN10 cells transformed with pS1 (empty vector); No. 2, pS1-full-length GA; No. 3, pS1-GAΔ26–167; No. 4, pS1-GAΔ132–167; No. 5, pS1-T165A and No. 6, pS1-N167D. (D) The electrophoretic mobilities of wild-type GA (lane 1) and N167D (lane 2) on an 8% SDS-PAGE.

The minimal active linker region Ala^161 ^to Asn^167 ^was previously demonstrated to be extremely important for GA function. In addition, N-terminal sequencing analysis of the GAΔ26–160 yielded the following sequence: Ala^161^-Thr^162^-Phe^163^-Pro^164^-Xaa^165-^Gly^166^-Xaa^167^, where Xaa represented unidentified residues. Generally speaking, biochemically modified amino acid residues were not identifiable by regular Edman degradation; hence residues Thr^165 ^and Asn^167 ^were possibly modified after translation. To abolish the glycosylation and evaluate the consequence, Thr^165 ^and Asn^167 ^in the linker region were individually mutated to generate T165A and N167D, respectively, and their effects on protein secretion and enzymatic activity were examined at 30°C. T165A mutation showed no effect on the phenotype as compared with wild-type full-length GA (Figure 4A and 4B, upper panel, lanes 2 and 5), while a significant decrease in GA expression and function was observed in the N167D mutant (Figure 4A and 4B, upper panel, lane 6). Site-specific mutation at Asn^167 ^led to much less active GA secretion, presumably due to either misfolding of *Ro*GA, or faster degradation of the nascent protein. In support of the misfolding hypothesis, several different methods were used to improve the production of the mutant GA, N167D. One evidence showed that N167D exhibited enhanced expression at 20°C (Figure 4B, lower panel, lane 6), and about 0.5 mg protein could be obtained from each liter of yeast culture at 20°C. The activity of the N167D mutant could also be detected directly on starch-agar plate with prolonged incubation at 4°C (Figure 4C, No. 6). Similar phenomena were also observed for the mutants GAΔ132–167 and GAΔ26–167 after prolonged incubation at low temperature (Figure 4C, No. 3 and No. 4).

Moreover, elimination of the N-glycosylation site at position Asn^167 ^(N167D) resulted in a faster relative electrophoretic mobility and lower apparent molecular weight (Figure 4D), providing additional line of evidence that the only putative N-glycosylation site in the linker region, Asn^167^-Ser-Thr, was indeed modified in *S. cerevisiae*. The biological function of carbohydrates in GA is not, however, completely understood, although it directs protein folding, facilitates secretion, and enhances stability of *Aspergillus *GAs [24]. In this study, we have demonstrated that N-glycosylation at Asn^167 ^in the linker is required for functional expression of *Ro*GA, whereas mutation abolishing N-glycosylation in the linker, N167D, decreased the efficiency of protein expression. Furthermore, similar result was obtained when Asn^167 ^was converted to Gln (data not shown). N-linked carbohydrates play important roles for a variety of structural and functional activities of glycoproteins [40]. Here, we have characterized the N-glycans attached to the linker region of *Ro*GA to be important for folding process, especially for its catalytic domain.

### The starch-binding domain

**Figure 5:**
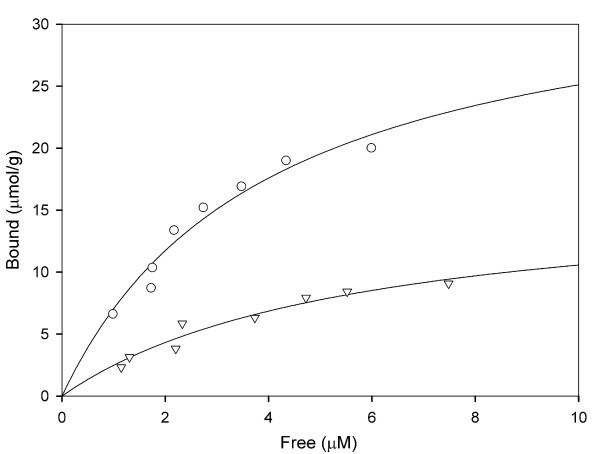
**Binding of purified SBD to corn-starch**. SBD, at different concentrations, was allowed to adsorb to insoluble corn-starch (1 mg) to the point of equilibrium. Protein concentrations were determined by direct measurement of unbound protein in solution. The data shown are representative of three independent experiments. Symbols: GAΔ132–604 (open circle) and GAΔ168–604 (open triangle).

The SBD, the non-catalytic module of GA that binds raw starch [21], is found at the N terminus in *Ro*GA and shares only 13.5% identity with that of *An*GA [22]. Characterization of the key functional groups of this domain has been accomplished using sequence-based structure alignment and NMR spectroscopy [22,23]. The effects of the linker region on SBD adsorption on to insoluble starch were thus investigated. The binding assay was conducted at pH 4.5 using insoluble corn-starch as the affinity matrix. Figure 5 showed the binding isotherms for the interaction between corn-starch and the SBD variants (GAΔ132–604 and GAΔ168–604). The binding isotherms were used to calculate binding parameters as described in the Materials and Methods section. The *K*_d _values for GAΔ132–604 and GAΔ168–604 were determined to be 3.98 μM and 5.99 μM, respectively; and the *B*_max _values of GAΔ132–604 and GAΔ168–604 were measured as 35.12 μmol/g and 17.24 μmol/g, respectively. It was apparent that the 106-residue SBD, GAΔ132–604, possessed stronger ligand affinity and higher capacity than those of the 142-residue SBD, GAΔ168–604, indicating that the presence of the linker region in the SBD construct did not increase but instead slightly interfered with the raw starch-binding affinity. The difference in starch binding affinity between GAΔ132–604 and GAΔ168–604 might be due to higher sugar content in the linker region or steric hindrance caused by the linker tail. Binding of the starch-degrading enzyme to its substrate is a critical step in starch hydrolysis because it involves the phase transfer of a soluble enzyme to the insoluble substrate [15]. In our case the linker sequence influences the binding of the SBD to starch, possibly by affecting the ligand transfer process.

### The catalytic domain

GA activity was assayed by measuring the reducing sugar released from the reaction on starch. The standard assay was performed as previously described with minor modification [17]. The specific activities of the wild-type *Ro*GA and the truncated mutants are listed in Table 3. Full-length GA and GAΔ26–131 exhibited similar activities, 4.58 × 10^3 ^U/μmol and 4.88 × 10^3 ^U/μmol, respectively, indicating that the absence of the N-terminal SBD had little or no effect on the ability to digest soluble starch. Moreover, comparison of the activities of the three catalytic domain variants GAΔ26–131, GAΔ26–145 and GAΔ26–160 revealed that longer size of the linker region was correlated with enhanced catalytic activity.

**Figure 6:**
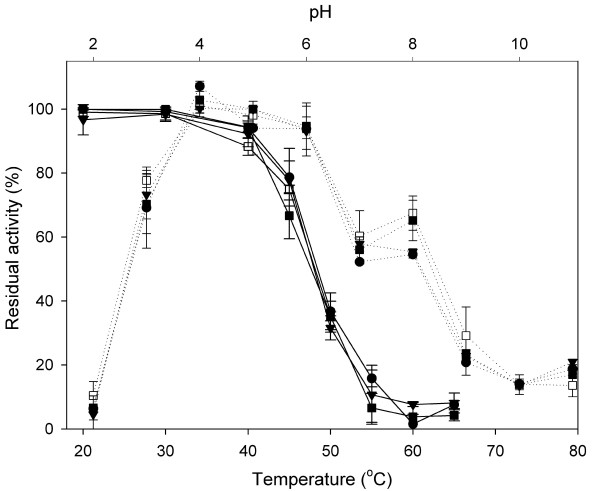
**Effects of temperature and pH on the enzymatic activity of *Ro*GA**. The effect of temperature on enzymatic activity (solid curves) was determined by pre-incubating the enzyme at pH 4.5 for 30 min at different temperatures and then assayed for residual activity. The effect of pH on GA activity (dotted curves) was performed in 0.1 M glycine/acetate/phosphate/tris buffers at the indicated pH values at 25°C. The activities measured without any pretreatment were defined as 100%. Each data point represents the average of three measurements, and error bars represent the S.D. Symbols: full-length GA (open square), GAΔ26–131 (closed circle), GAΔ26–145 (closed triangle), and GAΔ26–160 (closed square).

Figure 6 showed the effects of temperature and pH on enzyme activity. Regarding the thermal stability, the full-length *Ro*GA remained stable at 40°C for 30 min at pH 4.5 with almost 100% of its activity remained, whereas at the same pH only approximately 35% and 5% residual activity was respectively detected at 50°C and 60°C (Figure 6, solid curve, open square). The three catalytic domain derivatives possessed inactivation profiles similar to that of the full-length GA (Figure 6, solid curves), indicating that the linker region did not contribute as much as expected to the thermal stability. In addition, the pH stability of the full-length *Ro*GA at 25°C was found to be quite high over the pH range between 4.0 and 6.0, and more than 70% and 50% of the activity remained after 2 h incubation (Tm, 25°C) at pH 3.0 and pH 8.0, respectively (Figure 6, dotted curves). The truncated enzymes showed similar trends in stability over the pH range tested, indicating that the linker region was not crucial for the pH stability of GA either. Our data imply that the catalytic domain can function independently in terms of digesting soluble starch and the addition of linker sequences does not affect the thermal and pH stabilities of the enzyme.

**Table 3:** Specific activity of wild-type *Ro*GA and truncated mutants. Specific enzyme activities were evaluated per micromole of protein.

**Enzyme**	**Specific activity (10**^**3 **^U/μmol)*
full-length *Ro*GA	4.58 ± 0.16
GAΔ26–131	4.88 ± 0.04
GAΔ26–145	4.05 ± 0.06
GAΔ26–160	3.73 ± 0.33

*The data are expressed as the means ± SD.

### Structure analysis

**Figure 7:**
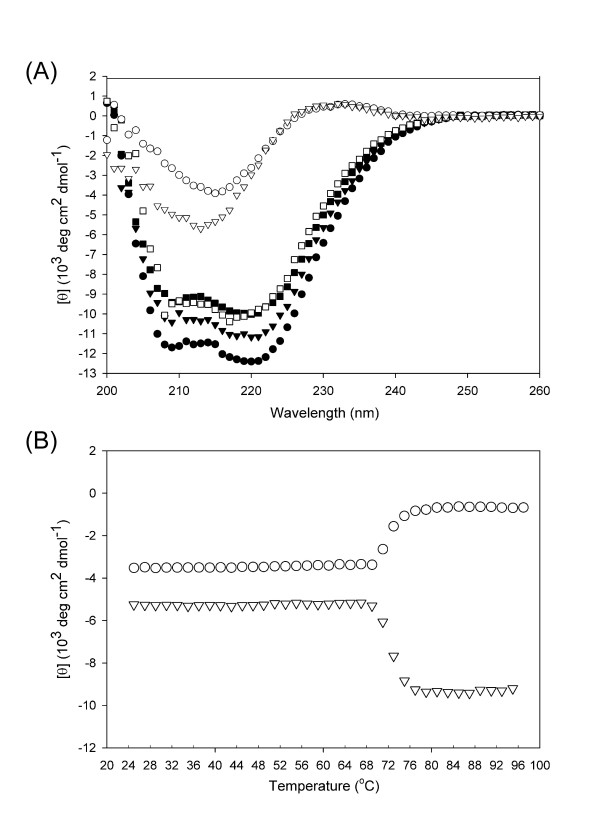
**Structural determination of wild-type and mutant *Ro*GAs**. (A) Each circular dichroism spectrum represents an average of three scans. The data were recorded at 25°C and corrected for the baseline contribution of the buffer. Spectra were truncated below 200 nm because of excessive noise at those wavelengths. Symbols: full-length GA (open square), GAΔ132–604 (open circle), GAΔ168–604 (open triangle), GAΔ26–131 (closed circular), GAΔ26–145 (closed triangle) and GAΔ26–160 (closed square). (B) Thermal denaturation curves of GAΔ132–604 (open circle) and GAΔ168–604 (open triangle) were obtained by monitoring changes in ellipticity at 215 nm.

The three-dimensional structure of *Ro*GA is currently unavailable; circular dichroism spectroscopy has been widely used to study the secondary structure, with the goal of understanding the role of the linker in protein structure. In this study, the far-UV spectrum of full-length *Ro*GA displayed two broad minima at 208 and 222 nm, characteristic of the presence of a mainly α-helical structure (Figure 7A, open square). The circular dichroism spectra of catalytic domain variants with different linker lengths were also indicative of a high content of helical conformation. In addition, the spectra of GAΔ26–131, GAΔ26–145, and GAΔ26–160 were very similar to each other, mainly differing in absorbance intensity (Figure 7A, closed symbols). GAΔ26–131 possessed a higher fraction of ordered secondary structure than did the other mutants, suggesting that the linker sequence might increase the stability of the secondary structural motifs. It was thus concluded that the linker sequences or their high degree of glycosylation facilitated stabilization of conformation of the catalytic domain of *Ro*GA.

As for the SBD, Figure 7A also showed the calculated circular dichroism spectra of GAΔ132–604 and GAΔ168–604 (open triangle and circle, respectively). The negative ellipticity peak centered at 215 nm indicated that the secondary structure of the protein was predominantly β-sheet, but the latter showed a higher β-sheet conformation content. Similar to the case for the catalytic domain variants, the presence of the linker peptide in the recombinant SBD induced a substantial conformational change to a more ordered state. In addition, the thermal denaturation of GAΔ132–604 and GAΔ168–604 was monitored by circular dichroism at 215 nm. Figure 7B revealed that the denatured state of GAΔ168–604 possessed a greater negative ellipticity at 215 nm than did GAΔ132–604, suggesting that the presence of linker sequence might induce the SBD to form a more compact structure at high temperature. Taken together, although the 36-amino-acid linker alone of *Ro*GA is predicted to be disordered, it strongly influences the structural ordering of the independent functional domains of *Ro*GA.

## Conclusion

Many raw starch-degrading enzymes have a modular structure in which the functional domains are spatially separated by linker sequences. In *An*GA, the highly O-glycosylated linker segment has been shown to be needed for the efficient digestion of raw starch [24,41]. In contrast, the raw starch-digesting α-amylase from the yeast *Cryptococcus *sp. strain S-2, lacking of a linker segment demonstrates that the linker region is not always essential for the discrete domains in raw starch-degrading enzymes to retain their proper function [42]. Our present study focuses on functional characterization of the linker region of *Ro*GA. The linker between the SBD and the catalytic domain consists of 36 amino acids with several oligosaccharides attached, and the region facilitates the formation of functional *Ro*GA in *S. cerevisiae*. The GAΔ132–167 deletion mutant failed to produce detectable protein at 30°C, presumably because the distance between the two domains was too short for them to be folded and secreted correctly. Interestingly, prolonged incubation at low temperature was allowed to synthesize the mutant, which showed detectable activity as visualized on starch plate (Figure 4C No. 3), indicating that low temperature might work in concert to increase the *de novo *synthesis and stabilize correct folding of the mutant protein. In addition, our results show that the properties of two functional domains are influenced by the linker region. The linker sequence influences the substrate binding process, possibly in the substrate transfer step. Analysis of the isolated catalytic domain shows that the formation of functional domain requires the linker region, especially the amino acid sequences from 161 to 167. Interestingly, the single N-glycosylation site within the linker domain, Asn^167^, is demonstrated to be extremely important for the function of *Ro*GA. Some glycoproteins need the N-glycans during synthesis for proper folding, sufficient for the efficient secretion or increased stability [43-45]. In this report, we have demonstrated that the N-linked oligomannosides in the linker region play an important role in expression of functional *Ro*GA, however, the exact structures of the oligosaccharides attached to the linker segment remain to be further investigated.

Prior to our present work, no three dimensional structure of intact *Ro*GA has ever been reported. The catalytic domain of *Ro*GA shows 36% identity and 52% similarity to that of *An*GA, a functional domain with a characteristic α-helical structure. As for the three dimensional structure of SBD, it has been initially molecular modeled by structural bioinformatics methodology [22] and subsequently determined by NMR spectroscopy [23]. Interestingly, while the SBDs of *Ro*GA and *An*GA share extremely low similarity in their primary structures, their secondary and tertiary structures appear to be quite similar, strongly suggesting that SBDs from CBM20 and CBM21 can be grouped into a new clan with similar functional structures [27,28]. It has been clearly demonstrated that the presence of the linker region leads to much more stable conformation of the two discrete functional domains. Taken together, our data provide several lines of direct evidence that the linker region of *Ro*GA plays a crucial role in terms of holding structural integrity, enhancing structure stability, and facilitating functional protein expression.

## Methods

### Strains, media, and expression plasmid

*Escherichia coli *TOP10F' (Invitrogen) was used for plasmid manipulations, and the *S. cerevisiae *strain MNN10 (*MAT*a, *mnn*10, *leu*2Δ0, *his*3Δ1, *met*15Δ0, *ura*3Δ0) was used for protein expression. *E*. *coli *cells were grown in Luria-Bertani medium (1% tryptone, 0.5% yeast extract, and 0.5% sodium chloride) at 37°C. Yeast cultures were grown in YPD (1% yeast extract, 2% peptone, and 2% glucose) or synthetic minimal (SD) medium (0.67% yeast nitrogen base supplemented with the appropriate amino acids and 2% fructose) at 20 or 30°C. The expression vector used in this work was pYEX-S1 (pS1, Clontech), a yeast-*E*. *coli *shuttle vector containing the phosphoglycerate kinase (PGK) promoter, the *E*.*coli *Amp^r ^gene, and the yeast selectable marker *URA*3.

### Construction of the full-length RoGA construct

The full-length cDNA encoding *Ro*GA was kindly provided by Simpson Biotech, and the set of primers F-*Ro*GA and R-*Ro*GA was used to clone the *Ro*GA gene. Both primers included an additional *Eco*RI site at their 5'-ends. The sequences of all the primers used are listed in Table 4. PCR amplification was carried out with KlenTaq-1 DNA polymerase (Clontech). The 1.8-kb PCR product was gel-purified and subcloned into yT&A TA cloning vector (Yeastern Biotech) to generate a pT-*Ro*GA plasmid, which was further digested with *Eco*RI and then subcloned into pS1 at the same enzyme site to generate a pS1-*Ro*GA plasmid.

**Table 4:** Oligonucleotide primers used for the construction of plasmids. Primers were used in the amplification reactions to generate various DNA fragments or mutations constructs.

**Name**	Nucleotide sequence*
F-*Ro*GA	5'-TTCGAATTCATGCAATTATTCAATTTG-3'
R-*Ro*GA	5'-TTCGAATTCTTAAGCGGCAGGTGCACC-3'
R-GAΔ168–604	5'-TTGAATTCTCAGTTACCAGTTGGGA-3'
R-GAΔ132–604	5'-TTGAATTCTCATGTAGATACTTGGT-3'
F-PS1	5'-CGTAGTTTTTCAAGTTCTTAG-3'
R-PS1	5'-TCCTTACCTTCCAATAATTC-3'
F-GAΔ132–167	5'-CAAGTATCTACATCTACAATCTCC-3'
R-GAΔ132–167	5'-AGGAGATTGTAGATGTAGATACTTG-3'
F-GAΔ26–131	5'-TTGCTTGTTTCTGCTTCCAAGCCCACTACT-3'
R-GAΔ26–131	5'-AGTAGTGGGCTTGGAAGCAGAAACAAGCAA-3'
F-GAΔ26–145	5'-AGAAGTAGAAGGGGCAGCAGAAACAAGCAA-3'
R-GAΔ26–145	5'-TTGCTTGTTTCTGCTGCCCCTTCTACTTCT-3'
F-GAΔ26–160	5'-TTGCTTGTTTCTGCTGCTACTTTCCCAACT-3'
R-GAΔ26–160	5'-AGTTGGGAAAGTAGCAGCAGAAACAAGCAA-3'
F-GAΔ26–167	5'-TTGCTTGTTTCTGCTTCTACAATCTCCTCA-3'
R-GAΔ26–167	5'-TGAGGAGATTGTAGAAGCAGAAACAAGCAA-3'
F-T165A	5'-CCA**GCT**GGTAACTCTACAATCTCCTCA
R-T165A	5'-GGAGATTGTAGAGTTACC**AGC**TGG
F-N167D	5'-ACTGGT**GAC**TCTACAATCTCCTCATGGATT-3'
R-N167D	5'-GGAGATTGTAGA**GTC**ACCAGTTGGGAAAGT-3'

*Underlined letters indicate *Eco*RI sites. Bold letters indicate mutated nucleotides.

### Construction of deletion mutants of RoGA

A series of deletion mutants was generated by a PCR-based technique using the pS1-*Ro*GA plasmid as the template. For the C-terminal truncation clone GAΔ168–604, which lacks the catalytic domain, the template was amplified using the primer pair F-*Ro*GA and R-GAΔ168–604. For GAΔ132–604, which lacks both the linker region and the catalytic domain, the template was amplified using primers F-*Ro*GA and R-GAΔ132–604. The resulting PCR products were separated on a 1% agarose gel and subcloned into pS1 as described above. The internal deletion clone GAΔ132–167, which lacks the entire linker sequence, was generated by fusing an N-terminal fragment to a C-terminal fragment with a two-step PCR approach. In the first step, F-PS1 paired with R-GAΔ132–167 and F-GAΔ132–167 paired with R-PS1 were used to generate PCR products of 516 bp and 1517 bp, respectively. The PCR products thus obtained shared overlapping sequences that could anneal in a secondary PCR. In the second step, the two first-stage PCR products were purified, combined and reamplified together with the external primers F-PS1 and R-PS1. The generated PCR product was subcloned into pS1 and sequenced for confirmation. Similar technologies were employed to obtain the other internal deletion clones with the following primer pairs: GAΔ26–131, F-GAΔ26–131 and R-GAΔ26–131; GAΔ26–145, F-GAΔ26–145 and R-GAΔ26–145; GAΔ26–160, F-GAΔ26–160 and R-GAΔ26–160; GAΔ26–167, F-GAΔ26–167 and R-GAΔ26–167. All primer sequences are listed in Table 4.

### Halo assay for GA activity

*S. cerevisiae *transformants were patched on YPD agar plate containing 0.5% (w/v) soluble starch. Secreted GA activity was detected by observing halo formation on agar plates containing iodine solution (0.01%).

### Purification of secreted proteins from S. cerevisiae

Yeast strains were transformed using the one-step transformation method as described [46]. The transformed yeast was cultivated in SD medium for 3 days at 30°C, and the supernatant of the culture medium was concentrated and dialyzed against 10 mM NaOAc (pH 4.5) using an Amicon stirred-cell concentrator (Millipore) equipped with a PM-10 membrane (10-kDa cut-off). For purification, the concentrated sample was loaded onto a 5-mL HiTrap SP cation-exchange column (Amersham Pharmacia Biotech) that was pre-equilibrated with the same buffer used for dialysis. The column was washed with 5 column volumes of 10 mM NaOAc buffer and then eluted with 10 volumes of a linear gradient of NaCl from 0 to 1 M in the same buffer at a flow rate of 2 mL/min. Fractions in 1-mL volumes were collected, and protein peaks were monitored by UV absorption at 280 nm. The fractions containing the desired proteins were pooled, dialyzed, and concentrated. The protein concentration was determined using the bicinchoninic acid (BCA) protein assay reagent kit (Pierce), with BSA as the reference standard.

### Electrophoresis and Western blot analysis

SDS-PAGE was performed according to the method of Laemmli [47] using 10 or 15% (w/v) polyacrylamide gels. The protein bands in the gel were revealed by staining with Coomassie Brilliant Blue R-250. In Western blot analysis, extra- and intra-cellular samples were separated by SDS-PAGE and transferred electrophoretically onto PVDF membranes. The resulting Western blots were incubated with 3% BSA in TBS as the blocking solution and probed with polyclonal anti-*Ro*GA antibody, used at a 1:5000 dilution. The secondary antibody was the horseradish peroxidase-conjugated anti-rabbit IgG, diluted to 1:5000. The bound complexes were detected with ECL reagents (ECL kit, Pierce) and exposure to X-ray film.

### Mass spectrometric analysis

Molecular mass determination of the recombinant proteins was performed by Liquid Chromatography/Mass Spectrometer (LC/MS). The intact proteins (100 pmol) were acidified with 0.1% formic acid in 50% (v/v) acetonitrile, and the data were acquired over the 800–1800 *m/z *range under normal scan resolution. The original electrospray mass spectrum with multiply charged ion series was deconvoluted to give a mass spectrum.

### N-terminal peptide sequence analysis

For N-terminal amino acid sequencing, purified protein was separated by SDS-PAGE, and electrophoretically transferred to a PVDF membrane. The blotted proteins were visualized using Coomassie Brilliant Blue R-250. After destaining, the blots were rinsed in deionized water and air-dried. N-terminal sequencing was performed by automated Edman degradation using an Applied Biosystems model 494 Procise sequencer at the National Taiwan University.

### Deglycosylation assays

Peptide N-glycosidase F (PNGase F, New England Biolabs) and Jack bean α-mannosidase (Sigma-Aldrich) were used in enzymatic deglycosylation of the recombinant proteins according to the manufacturers' instructions. After enzymatic treatment, the samples were separated by SDS-PAGE and stained with Coomassie Brilliant Blue R-250 to detect any differences in protein migration.

### Site-directed mutagenesis of amino acid residues in the linker

Site-directed mutagenesis was performed with a PCR-based technique to change Thr^165 ^to Ala and Asn^167 ^to Asp. The primers used were as follows: universal primers, F-PS1 and R-PS1; mutagenic primers F-T165A and R-T165A for the Thr^165^→Ala substitution; and F-N167D and R-N167D for the Asn^167^→Asp substitution. All primer sequences are listed in Table 4.

### Adsorption assay

The adsorption of the SBD to granular corn-starch (Sigma-Aldrich) was performed as follows. The purified SBD-containing protein at a concentration ranging from 1.0 to 7.5 μM was mixed with 1 mg of prewashed corn-starch in a final volume of 1 mL in 10 mM NaOAc, pH 4.5. After incubation at 25°C for 16 h, the starch was removed by centrifugation at 16,000 *g *for 3 min at 4°C, and the amount of unbound protein remaining in the supernatant was determined by the BCA assay. The amount of adsorbed protein was calculated from the difference between the initial and unbound protein concentrations. The maximal amount of bound protein (*B*_max_) and the dissociation constant (*K*_d_) were determined by fitting to the non-linear regression of the binding isotherms, and the following equation was used for saturation binding with one binding site: *B *= *B*_max_*F*/(*K*_d_+*F*), where *B *(μmol) represents the bound protein, *B*_max _(μmol) is the maximal amount of bound protein, *F *(μmol) is the free protein in the system, and *K*_d _(μmol) is the equilibrium dissociation constant. The units of calculated *B*_max _and *K*_d _were converted to micromoles per gram and micromolar, respectively.

### GA activity toward soluble starch

Substrate was prepared by boiling 1% soluble corn-starch in 10 mM NaOAc, pH 4.5. The amount of glucose liberated from the starch by enzymatic activity was determined using the glucose oxidase/peroxidase kit (Sigma-Aldrich). For each reaction, 50 μL of substrate solution was equilibrated in a 37°C water bath for 5 min, and the assay was initiated by adding 50 μL of appropriately diluted enzyme solution. After incubation at 37°C for 5 min, 200 μL of kit solution was added to each sample. The reaction mixtures were incubated for 5 min at 37°C and stopped by the addition of 200 μL of 6 M H_2_SO_4_. The absorbance was recorded at 540 nm using a U-3310 spectrophotometer (Hitachi). One unit of enzyme activity was defined as the amount of enzyme that produces 1 μmol glucose/min under the assay conditions described above.

### Effect of temperature and pH on the activity of GA

Thermal stability of the enzyme was determined by pre-incubating the purified protein for 30 min at various temperatures ranging from 20 to 65°C. At time intervals, the residual activity was measured with the glucose assay kit as described above. Stability of the recombinant protein at different pH values was studied by incubating the enzyme in various buffers with pH values ranging from 2.0 to 11.0 for 2 h and then measuring the residual GA activity. The buffers used were 0.1 M glycine-HCl (pH 2.0, 3.0), 0.1 M sodium acetate (pH 4.0, 5.0), 0.1 M potassium phosphate (pH 6.0, 7.0), 0.1 M Tris-HCl (pH 8.0), and 0.1 M glycine-NaOH (pH 9.0, 10.0, 11.0).

### Circular dichroism studies

Circular dichroism measurements were carried out with an AVIV model 202 spectropolarimeter (Aviv Associates, Lakewood, NJ). Far-UV wavelength scans were recorded from 200 to 260 nm with a bandwidth of 1.0 nm, using a 0.1-cm path length cuvette at 25°C. Each spectrum was an average of three consecutive scans and was corrected by subtracting the buffer spectrum. Thermal denaturation experiments were performed by increasing the temperature from 25 to 96°C, allowing temperature equilibration for 1.5 min before recording each spectrum. All of the data are expressed in terms of mean residue ellipticity, [*θ*]_m.r.w._, calculated by the equation: [*θ*]_m.r.w. _= (100*θ*_obs_)/(*nlc*), where *θ*_obs _is the observed ellipticity in degrees, *n *is the number of amino acids, *l *is the length of the light path in centimeters, and *c *is the molar concentration of the protein.

## Abbreviations

*An *GA, *Aspergillus niger *glucoamylase; *Ro *GA, *Rhizopus oryzae *glucoamylase; CBM, carbohydrate-binding module; SBD, starch-binding domain

## Authors' contributions

SCL designed and generated most of the mutants, carried out most functional assays, and drafted the manuscript; WTL expressed and purified recombinant SBDs. SHL involved initial cloning and enzymatic assay designs; WIC participated in the structural determination employing spectroscopic methodologies; BKH assisted recombinant yeast generation and growth. IPL participated in manuscript preparation. CCS participated in experimental design and coordination. MDTC conceived and supervised this project and manuscript writing. All authors read and approved the final manuscript.

## References

[B1] Hiromi K, Hamauzu ZI, Takahashi K, Ono S (1966). Kinetic studies on gluc-amylase. II. Competition between two types of substrate having alpha-1,4 and alpha-1,6 glucosidic linkage. J Biochem (Tokyo).

[B2] Sauer J, Sigurskjold BW, Christensen U, Frandsen TP, Mirgorodskaya E, Harrison M, Roepstorff P, Svensson B (2000). Glucoamylase: structure/function relationships, and protein engineering. Biochim Biophys Acta.

[B3] Norouzian D, Akbarzadeh A, Scharer JM, Moo Young M (2006). Fungal glucoamylases. Biotechnol Adv.

[B4] Sakai Y, Akiyama M, Kondoh H, Shibano Y, Kato N (1996). High-level secretion of fungal glucoamylase using the Candida boidinii gene expression system. Biochim Biophys Acta.

[B5] Manjunath P, Shenoy BC, Raghavendra Rao MR (1983). Fungal glucoamylases. J Appl Biochem.

[B6] Coutinho PM, Reilly PJ (1997). Glucoamylase structural, functional, and evolutionary relationships. Proteins.

[B7] Mertens JA, Skory CD (2007). Isolation and Characterization of Two Genes That Encode Active Glucoamylase Without a Starch Binding Domain from Rhizopus oryzae. Curr Microbiol.

[B8] Aleshin A, Golubev A, Firsov LM, Honzatko RB (1992). Crystal structure of glucoamylase from Aspergillus awamori var. X100 to 2.2-A resolution. J Biol Chem.

[B9] Chen HM, Ford C, Reilly PJ (1994). Substitution of asparagine residues in Aspergillus awamori glucoamylase by site-directed mutagenesis to eliminate N-glycosylation and inactivation by deamidation. Biochem J.

[B10] Libby CB, Cornett CA, Reilly PJ, Ford C (1994). Effect of amino acid deletions in the O-glycosylated region of Aspergillus awamori glucoamylase. Protein Eng.

[B11] Semimaru T, Goto M, Furukawa K, Hayashida S (1995). Functional analysis of the threonine- and serine-rich Gp-I domain of glucoamylase I from Aspergillus awamori var. kawachi. Appl Environ Microbiol.

[B12] Chen L, Coutinho PM, Nikolov Z, Ford C (1995). Deletion analysis of the starch-binding domain of Aspergillus glucoamylase. Protein Eng.

[B13] Sorimachi K, Le Gal-Coeffet MF, Williamson G, Archer DB, Williamson MP (1997). Solution structure of the granular starch binding domain of Aspergillus niger glucoamylase bound to beta-cyclodextrin. Structure.

[B14] Sauer J, Christensen T, Frandsen TP, Mirgorodskaya E, McGuire KA, Driguez H, Roepstorff P, Sigurskjold BW, Svensson B (2001). Stability and function of interdomain linker variants of glucoamylase 1 from Aspergillus niger. Biochemistry.

[B15] Paldi T, Levy I, Shoseyov O (2003). Glucoamylase starch-binding domain of Aspergillus niger B1: molecular cloning and functional characterization. Biochem J.

[B16] Goto M, Shinoda N, Oka T, Sameshima Y, Ekino K, Furukawa K (2004). Thr/Ser-rich domain of Aspergillus glucoamylase is essential for secretion. Biosci Biotechnol Biochem.

[B17] Liu SH, Chou WI, Sheu CC, Chang MD (2005). Improved secretory production of glucoamylase in Pichia pastoris by combination of genetic manipulations. Biochem Biophys Res Commun.

[B18] Liu SH, Chou WI, Lin SC, Sheu CC, Chang MD (2005). Molecular genetic manipulation of Pichia pastoris SEC4 governs cell growth and glucoamylase secretion. Biochem Biophys Res Commun.

[B19] Tanaka Y, Ashikari T, Nakamura N, Kiuchi N, Shibano Y, Amachi T, Yoshizumi H (1986). Comparison of amino acid sequences of three glucoamylases and their structure-function relationships. Agric Biol Chem.

[B20] Cornett CA, Fang TY, Reilly PJ, Ford C (2003). Starch-binding domain shuffling in Aspergillus niger glucoamylase. Protein Eng.

[B21] Rodriguez-Sanoja R, Oviedo N, Sanchez S (2005). Microbial starch-binding domain. Curr Opin Microbiol.

[B22] Chou WI, Pai TW, Liu SH, Hsiung BK, Chang MD (2006). The family 21 carbohydrate-binding module of glucoamylase from Rhizopus oryzae consists of two sites playing distinct roles in ligand binding. Biochem J.

[B23] Liu YN, Lai YT, Chou WI, Chang MD, Lyu PC (2007). Solution structure of family 21 carbohydrate-binding module from Rhizopus oryzae glucoamylase. Biochem J.

[B24] Goto M, Tsukamoto M, Kwon I, Ekino K, Furukawa K (1999). Functional analysis of O-linked oligosaccharides in threonine/serine-rich region of Aspergillus glucoamylase by expression in mannosyltransferase-disruptants of yeast. Eur J Biochem.

[B25] Janecek S, Svensson B, MacGregor EA (2003). Relation between domain evolution, specificity, and taxonomy of the alpha-amylase family members containing a C-terminal starch-binding domain. Eur J Biochem.

[B26] Henrissat B (1991). A classification of glycosyl hydrolases based on amino acid sequence similarities. Biochem J.

[B27] Machovic M, Svensson B, MacGregor EA, Janecek S (2005). A new clan of CBM families based on bioinformatics of starch-binding domains from families CBM20 and CBM21. FEBS J.

[B28] Machovic M, Janecek S (2006). Starch-binding domains in the post-genome era. Cell Mol Life Sci.

[B29] Denman S, Xue GP, Patel B (1996). Characterization of a Neocallimastix patriciarum cellulase cDNA (celA) homologous to Trichoderma reesei cellobiohydrolase II. Appl Environ Microbiol.

[B30] Howard MB, Ekborg NA, Taylor LE, Hutcheson SW, Weiner RM (2004). Identification and analysis of polyserine linker domains in prokaryotic proteins with emphasis on the marine bacterium Microbulbifer degradans. Protein Sci.

[B31] Dell A, Morris HR (2001). Glycoprotein structure determination by mass spectrometry. Science.

[B32] NetNGlyc http://www.cbs.dtu.dk/services/NetNGlyc/.

[B33] Ash J, Dominguez M, Bergeron JJ, Thomas DY, Bourbonnais Y (1995). The yeast proprotein convertase encoded by YAP3 is a glycophosphatidylinositol-anchored protein that localizes to the plasma membrane. J Biol Chem.

[B34] O'Leary JM, Radcliffe CM, Willis AC, Dwek RA, Rudd PM, Downing AK (2004). Identification and removal of O-linked and non-covalently linked sugars from recombinant protein produced using Pichia pastoris. Protein Expr Purif.

[B35] Jentoft N (1990). Why are proteins O-glycosylated?. Trends Biochem Sci.

[B36] NetOGlyc http://www.cbs.dtu.dk/services/NetOGlyc/.

[B37] Maley F, Trimble RB, Tarentino AL, Plummer TH (1989). Characterization of glycoproteins and their associated oligosaccharides through the use of endoglycosidases. Anal Biochem.

[B38] Zhang H, Li XJ, Martin DB, Aebersold R (2003). Identification and quantification of N-linked glycoproteins using hydrazide chemistry, stable isotope labeling and mass spectrometry. Nat Biotechnol.

[B39] De Praeter CM, Gerwig GJ, Bause E, Nuytinck LK, Vliegenthart JF, Breuer W, Kamerling JP, Espeel MF, Martin JJ, De Paepe AM, Chan NW, Dacremont GA, Van Coster RN (2000). A novel disorder caused by defective biosynthesis of N-linked oligosaccharides due to glucosidase I deficiency. Am J Hum Genet.

[B40] Panda A, Elankumaran S, Krishnamurthy S, Huang Z, Samal SK (2004). Loss of N-linked glycosylation from the hemagglutinin-neuraminidase protein alters virulence of Newcastle disease virus. J Virol.

[B41] Belshaw NJ, Williamson G (1990). Production and purification of a granular-starch-binding domain of glucoamylase 1 from Aspergillus niger. FEBS Lett.

[B42] Iefuji H, Chino M, Kato M, Iimura Y (1996). Raw-starch-digesting and thermostable alpha-amylase from the yeast Cryptococcus sp. S-2: purification, characterization, cloning and sequencing. Biochem J.

[B43] Imperiali B, Rickert KW (1995). Conformational implications of asparagine-linked glycosylation. Proc Natl Acad Sci USA.

[B44] Haraguchi M, Yamashiro S, Furukawa K, Takamiya K, Shiku H, Furukawa K (1995). The effects of the site-directed removal of N-glycosylation sites from beta-1,4-N-acetylgalactosaminyltransferase on its function. Biochem J.

[B45] Yanez E, Carmona TA, Tiemblo M, Jimenez A, Fernandez-Lobato M (1998). Expression of the Schwanniomyces occidentalis SWA2 amylase in Saccharomyces cerevisiae: role of N-glycosylation on activity, stability and secretion. Biochem J.

[B46] Chen DC, Yang BC, Kuo TT (1992). One-step transformation of yeast in stationary phase. Curr Genet.

[B47] Laemmli UK (1970). Cleavage of structural proteins during the assembly of the head of bacteriophage T4. Nature.

